# Functional and Cognitive Decline in an Older Adult With Severe Hyponatremia and Undiagnosed Hypothyroidism: A Geriatric Perspective

**DOI:** 10.7759/cureus.100682

**Published:** 2026-01-03

**Authors:** Parul Bhutani, Bhagyashree Tanwar, Nikhil Choudhary, Amit Singh, Minakshi Dhar

**Affiliations:** 1 Geriatric Medicine, All India Institute of Medical Sciences, Rishikesh, IND; 2 Geriatrics, All India Institute of Medical Sciences, Rishikesh, IND

**Keywords:** cognitive decline, dyselectrolytemia in elderly, functional decline, hyponatremia, hypothyroidism in elderly, older adult, reversible frailty

## Abstract

Hyponatremia is the most common electrolyte disturbance in older adults and often presents with nonspecific symptoms such as confusion, gait instability, and functional decline, frequently mistaken for ageing. Hypothyroidism is another reversible condition in the elderly that can impair free-water clearance and contribute to chronic slowing and cognitive dysfunction. We report an octogenarian man with chronic obstructive pulmonary disease, hypertension, and recently diagnosed diabetes who presented with one week of fluctuating altered mental status and hallucinations, superimposed on six months of progressive weakness and near-complete loss of independence. Evaluation revealed severe euvolemic hyponatremia (110 mEq/L), low serum osmolality, elevated urine sodium and osmolality, and marked hypothyroidism, with other causes such as syndrome of inappropriate antidiuretic hormone secretion and adrenal insufficiency excluded. He was treated with cautious hypertonic saline correction and levothyroxine. Over the following weeks, serum sodium and thyroid function normalized, accompanied by dramatic clinical recovery: cognition improved (Hindi Mental State Examination: 28/30), frailty decreased (Clinical Frailty Scale: 6 to 4), and independence in daily activities improved from 2/6 to 6/6. Unlike previous reports, this case demonstrates a geriatric viewpoint and near-complete reversal of geriatric syndrome and prolonged geriatric functional decline following correction of hypothyroidism-associated hyponatremia, highlighting the need to consider subtle endocrine abnormalities as fully reversible causes of decline in older adults.

## Introduction

Hyponatremia is one of the most common electrolyte disturbances encountered in older adults and is frequently associated with nonspecific symptoms such as gait instability, falls, lethargy, reduced oral intake, and cognitive impairment, even at mild levels of sodium reduction [1,2]. Chronic hyponatremia has been shown to impair attention, postural balance, and reaction time, increasing the risk of frailty, fractures, and functional decline in ageing populations [2,3]. Because these manifestations closely overlap with normal ageing and multimorbidity, the biochemical abnormality may remain undetected for extended periods.

Hypothyroidism is another under-recognized but reversible condition in older adults, with presentations often subtle and attributed to age-related slowing, depression, or neurocognitive disorders [4]. Long-standing hypothyroidism can contribute to fatigue, slowness, constipation, cognitive slowing, mood changes, and reduced performance in activities of daily living (ADLs) [4,5]. The association between hypothyroidism and hyponatremia remains debated; while impaired free-water clearance due to reduced glomerular filtration and inappropriate antidiuretic hormone activity has been proposed, the relationship is inconsistent across studies [6,7]. 

In frail older adults, even modest disturbances in serum sodium or thyroid function may precipitate significant decline because of reduced physiological reserve, polypharmacy, and multimorbidity. Comprehensive geriatric assessment (CGA) plays a crucial role in identifying potentially reversible contributors to functional and cognitive deterioration in such patients [8]. The relationship between thyroid dysfunction and cognitive decline in older adults remains complex, with studies showing mixed results [9,10].

We present a case of an older man with multimorbidity who developed progressive functional decline and acute cognitive deterioration. Severe hyponatremia in the context of unrecognized hypothyroidism was identified, with significant improvement following treatment. This case underscores the value of CGA in distinguishing reversible geriatric syndromes from irreversible age-related decline.

## Case presentation

An old man in his 80s, who is a chronic bidi smoker and a known case of chronic obstructive pulmonary disease (COPD) for the past 40 years, was on medication, including a long-acting muscarinic antagonist/long-acting beta-agonist inhaler. He also had hypertension for one year, controlled on amlodipine 5 mg. He was recently diagnosed with type 2 diabetes mellitus five months ago and started on oral hypoglycemic agents (metformin 500 mg once daily).

He presented to the emergency department with altered mental status for one week. One week prior to presentation, his caregivers noted acute fluctuating changes in his mental status, including episodes of confusion, irrelevant speech, visual hallucinations (calling out to people not present), poor attention, inability to recognize family members, disorientation to time and place, and impaired recent memory. These symptoms fluctuated during the day, with noticeable worsening in the evening, consistent with features of delirium. There was no recent history of fever, cough, chest pain, palpitations, shortness of breath, headache, weight loss, seizures, focal neurological deficits, recent trauma, vomiting, diarrhea, abdominal pain, medication changes, or substance abuse. After stabilization, he was shifted to the geriatric medicine ward for further treatment and evaluation.

A detailed history and examination were performed after admission. On repeated questioning, his son reported that he had complained of generalized weakness for the last five to six months and remained in bed most of the time. His ADL had been reduced to self-feeding and continence only, as he required the support of one person for all other basic activities. He had also been taken to multiple hospitals over the last six months, where he was diagnosed with type 2 diabetes mellitus; although it was controlled on oral medication, his weakness persisted. His son ultimately attributed his weakness to ageing alone and stopped pursuing further consultation for this.

On physical examination, he was not oriented to time, place, or person and was found to have delirium (Confusion Assessment Method positive). His Glasgow coma scale (GCS) was E4V5M6. Pulse rate was 66/min and regular; blood pressure was 134/60 mmHg; SpO₂ was 100% on room air; and temperature was 98.6°F. Clinical assessment showed features of euvolemia (moist mucous membranes, normal skin turgor, no peripheral oedema, normal jugular venous pressure, and stable hemodynamics). The rest of the systemic examination was unremarkable.

At admission, the patient was moderately frail (Clinical Frailty Scale (CSF): 6), dependent in most ADLs (Katz index for basic ADL: 2/6), at risk of malnutrition (Mini-Nutritional Assessment-Short Form (MNA-SF): 9), and screened positive for delirium, with physical and cognitive assessments not feasible due to poor general condition and delirious status of the patient (Table 1).

**Table 1:** CGA at admission, discharge, and follow-up OPD visit CSF: Clinical Frailty Scale; CAM: Confusion Assessment Method; MNA-SF: Mini-Nutritional Assessment-Short Form; HMSE: Hindi Mental State Examination; GDS: geriatric depression scale-15; TUG: timed up and go test; ADLs: activities of daily living; CGA: comprehensive geriatric assessment

Domain/tool	On admission	At discharge	Follow-up OPD visit
Basic ADLs (Katz index) [11]	2/6	4/6	6/6
CFS [12]	6 (moderate frailty)	5 (mild frailty)	4 (very mild frailty)
CAM [13]	Positive	Negative	Negative
MNA-SF [14]	9	10	-
HMSE [15]	Not feasible	28	28
GDS-15 [16]	Not feasible	3/15	3/15
TUG [17]	Not feasible	25 sec	20 sec
Hand grip strength [18]	Not feasible	28 kg	30 kg

He presented to our emergency department, where a non-contrast computed tomography (NCCT) scan of the head was performed in view of altered mental status, which revealed no acute bleed or infarct. His metabolic workup revealed severe hyponatremia (serum sodium 110 mEq/L) with low serum osmolality. Other metabolic causes were ruled out. As he had symptomatic severe hyponatremia, he was given an intravenous bolus of 100 mL of 3% NaCl in the emergency department and was then shifted to the Geriatric Medicine Ward. A clinical impression of euvolemic hyponatremia was made. Serum osmolality, urine sodium, urine osmolality, serum thyroid-stimulating hormone (TSH), and 8 am fasting serum cortisol were sent to evaluate the causes of euvolemic hyponatremia (Table 2).

**Table 2:** Investigation chart showing serial biochemical investigation trends TSH: thyroid-stimulating hormone; T3: triiodothyronine; T4: thyroxine (tetraiodothyronine)

Parameter (reference range)	14/02/25 emergency	15/02/25 admitted	16/02/25 admitted	18/02/25 admitted	07/03/25 OPD	29/03/25 OPD
TSH (0.4-4.0 µIU/mL)	-	23	-	-	15	3.5
Free T4 (0.8-1.8 ng/dL)	-	0.5	-	-	1.2	1.5
Free T3 (2.3-4.2 pg/mL)	-	2	-	-	2.4	4
Serum sodium (135-145 mEq/L)	110	120	122	125	128	137
Urine sodium (0-20 mEq/L)	-	74	-	-	-	-
Serum osmolality (275-295 mOsm/kg)	-	248	-	-	-	-
Urine osmolality (50-1200 mOsm/kg)	-	267	-	-	-	-
Potassium (3.5-5.5 mEq/L)	3.7	3.9	4.1	4.2	4.5	4.2
Urea (10-40 mg/dL)	20	22	-	-	-	20
Creatinine (0.5-1.0 mg/dL)	0.8	0.7	-	-	-	0.8
Hemoglobin (12-16 g/dL)	14	13.5	-	-	13	13.3
Total leukocyte count (4000-10000 cells/µL)	6000	6000	-	-	5300	5600
Platelet count (150000-350000 cells/µL)	104000	105000	-	-	135000	124000
Serum albumin (3.0-4.1 g/dL)	3.7	3.5	-	-	4.69	3.76
Serum cortisol (6-23 µg/dL)	-	14	-	-	-	-

Our first differential diagnosis was the syndrome of inappropriate antidiuretic hormone secretion (SIADH), which is the most common cause of euvolemic hyponatremia. A comprehensive review of his medications revealed no agents associated with SIADH. No evidence of pulmonary infection or malignancy, which could lead to SIADH, was found. Our second differential was glucocorticoid deficiency, which was ruled out by normal 8 am fasting cortisol levels. The third differential diagnosis for his euvolemic hyponatremia was hypothyroidism. His serum TSH level was elevated, with low total triiodothyronine (T3) and total thyroxine (T4) levels. Serum osmolality was reduced, and urine osmolality was elevated, with increased urine sodium and normal cortisol levels. Given the unexplained hyponatremia and chronic functional decline, along with elevated TSH (reference range: 0.4-4.0 µIU/mL) and low total T3 and T4 levels, a diagnosis of hypothyroidism-induced euvolemic hyponatremia was made. The etiology of hypothyroidism remained uncertain but was presumed to be chronic primary hypothyroidism.

Serum sodium correction was undertaken gradually, with daily increases maintained within recommended safety limits (<8 mmol/L per 24 hours) and close monitoring of serum sodium levels. Frequent electrolyte assessments were performed to avoid overcorrection, and no features of osmotic demyelination were observed during hospitalization or follow-up.

The patient was initiated on oral thyroxine supplementation and received supportive care for hyponatremia. Over the following days, his serum sodium gradually normalized in parallel with improvement in thyroid function. His mental status improved, and CAM screening became negative for two consecutive days prior to discharge.

At follow-up, repeat laboratory tests showed normal serum sodium and TSH levels (Table 2). The Comprehensive Geriatric Assessment demonstrated marked improvement across multiple domains from admission to follow-up, including functional status (B-ADL improved from 2/6 to 6/6), cognition (CAM turned negative; Hindi Mental State Examination (HMSE) 28/30), and frailty (CFS improved from 6 to 4) (Table 1).

These findings reflect significant recovery following treatment of the underlying reversible cause. Clinically, the patient reported improved energy levels and had regained independence in basic ADLs, even resuming short independent walks outdoors.

## Discussion

Just as the eyes are often described as windows to the brain, electrolyte imbalances can serve as windows to hidden systemic illnesses. In older adults, hyponatremia is the most common electrolyte disturbance and often presents insidiously, mimicking or exacerbating geriatric syndromes such as delirium, falls, and functional decline. These nonspecific presentations may lead clinicians to attribute symptoms to "normal ageing" rather than seek underlying pathology. However, each instance of hyponatremia in an elderly patient should prompt a thorough diagnostic workup, as it may unmask treatable conditions, such as hypothyroidism, as illustrated in our case. Recognizing and addressing such reversible causes can lead to remarkable recovery of physical function, cognition, and independence, significantly improving the patient's quality of life.

This case highlights the importance of considering endocrine causes in unexplained hyponatremia and functional deterioration among older adults. Hypothyroidism-related euvolemic hyponatremia is a recognized entity in textbooks but is rarely encountered in clinical practice, and the mechanisms through which hypothyroidism leads to hyponatremia are not fully understood. According to a recent review article by Chen et al. [1], hypothyroidism is thought to reduce cardiac output and increase peripheral vascular resistance, triggering baroreceptor-mediated release of arginine vasopressin (AVP). Additionally, mucopolysaccharide accumulation in interstitial tissues can decrease “effective” arterial blood volume, further stimulating AVP secretion. Both mechanisms enhance renal water reabsorption via aquaporin-2 channels, leading to dilutional hyponatremia. Furthermore, reduced cardiac output in hypothyroidism lowers the glomerular filtration rate (GFR), impairing the kidney’s ability to excrete free water and thereby diminishing sodium and water clearance, compounding water retention. Hypothyroidism may also decrease Na⁺/K⁺-ATPase activity in renal tubules, altering sodium reabsorption and subtly disrupting sodium balance, favoring dilutional hyponatremia [1].

Older patients may be particularly vulnerable due to age-related reductions in renal reserve and blunted water excretion ability. Although severe hyponatremia in hypothyroidism often occurs in cases like myxedema coma, milder hypothyroid-induced hyponatremia, especially chronic forms, is also seen in the elderly [1].

While previous case reports, such as those by Maharjan et al. (2022) [2] and Schmitz et al. (2001) [3], have demonstrated similar associations, our case is unique in presenting with profound geriatric syndromes and significant functional impairment that reversed following thyroxine therapy. Large studies like those by Chu et al. (2023) [4] and Ali et al. (2025) [5] have debated the frequency and causality of this association.

This patient’s six-month functional decline likely reflected chronic, untreated hypothyroidism, which is well documented to cause cognitive slowing, depressive symptoms, bradykinesia, fatigue, and reduced activity levels in older adults. Hypothyroidism is one of the most frequent endocrine disorders in later life, with prevalence increasing progressively with age. Population-based data (e.g., NHANES III) suggest an overall hypothyroidism prevalence of around 4%-5%, with subclinical disease far more common than overt disease and particularly frequent in older women [6,7]. In the “oldest old,” rising TSH concentrations and a higher burden of thyroid autoimmunity make biochemical hypothyroidism especially prevalent, although interpretation of thyroid function tests is complicated by comorbid illness, polypharmacy, and age-related shifts in reference ranges [6,7].

Clinical presentation in the elderly is often subtle and atypical. Rather than the classic symptoms described in younger adults, older patients may exhibit an “apathetic” form of hypothyroidism characterized by fatigue, cognitive slowing, depressive symptoms, constipation, hearing impairment, and vague functional decline, which are easily misattributed to normal ageing or coexisting multimorbidity [6,7]. Reviews focused on geriatric and critically ill populations highlight that hypothyroidism in older adults is associated with increased morbidity, falls, hospitalization, and, when untreated or undertreated, higher cardiovascular risk and mortality [6,7,11]. This background supports our decision to actively search for thyroid dysfunction in an octogenarian with otherwise unexplained cognitive and functional decline.

A growing body of work has explored the relationship between thyroid status and mobility, gait, and falls in older adults. In the Rotterdam Study, deviations in thyroid function were associated with altered gait patterns and poorer gait domains that are strongly linked to disability and fall risk [8]. Other cohort data suggest that both overt and, in some cases, subclinical hypothyroidism may influence walking speed and physical performance, though findings are not entirely consistent [7,8]. In our patient, the profound impairment in mobility and instrumental ADLs, followed by striking recovery after thyroxine replacement, is consistent with the concept that thyroid dysfunction can be a reversible contributor to sarcopenia, gait disturbance, and falls in the elderly.

The relationship between hypothyroidism and cognitive outcomes in older adults is complex. Systematic reviews and narrative syntheses indicate that overt hypothyroidism can cause potentially reversible cognitive impairment and mood changes, whereas data for subclinical hypothyroidism are heterogeneous, with several studies finding no clear association with global cognition, mood, or functional decline in very old individuals [7,9,10]. Nevertheless, even in the absence of definitive causal proof, many authors emphasize that thyroid dysfunction should be excluded in older adults presenting with new-onset cognitive impairment, depression, or apathy, because correction of overt hypothyroidism may lead to clinically meaningful cognitive and functional improvement in selected patients [6,7,9,10]. Our case adds to this body of evidence, illustrating that in an octogenarian with substantial functional and cognitive deterioration, targeted treatment of overt hypothyroidism was temporally associated with recovery of independence and mental status.

Management of hypothyroidism in the elderly is further complicated by frailty, cardiovascular comorbidities, and polypharmacy. Expert reviews recommend a cautious, individualized approach to levothyroxine initiation and titration in older adults, with lower starting doses and slower up-titration to minimize the risk of precipitating myocardial ischemia, arrhythmias, or bone loss [9,10]. At the same time, undertreatment may perpetuate fatigue, cognitive slowing, and functional dependency. Our patient’s course illustrates that, when thoughtfully implemented, thyroid hormone replacement can be safely delivered even in very old adults and can result in substantial gains in function and quality of life. This reinforces the importance of maintaining a high index of suspicion for hypothyroidism in elderly patients with unexplained functional decline, even when laboratory abnormalities (including hyponatremia) appear modest.

What distinguishes this case is not the rarity of hypothyroidism or hyponatremia in isolation, but the profound geriatric functional impairment that fully reversed once the underlying endocrine disorder was corrected. The patient had been dependent for most activities for six months and exhibited cognitive deterioration significant enough to prompt evaluation for dementia. Yet, following thyroxine replacement, he regained independence in mobility and basic ADLs, and cognitive scores improved substantially. Such dramatic functional recovery in an octogenarian after correction of a reversible metabolic cause underscores the importance of comprehensive geriatric assessment and diagnostic vigilance.

Hyponatremia in older adults is frequently multifactorial, and causality must be interpreted cautiously. Although the biochemical profile was consistent with euvolemic hyponatremia and hypothyroidism was identified as a reversible contributor, SIADH cannot be entirely excluded. Nevertheless, the temporal association between thyroid hormone replacement, gradual sodium correction, and sustained clinical improvement supports a significant contributory role of hypothyroidism in this case. We agree that the chronic decline was primarily driven by hypothyroidism and that hyponatremia may have been one of several downstream physiological consequences. Nonetheless, the presence of hyponatremia functioned as a valuable “signal abnormality” directing clinicians toward a treatable endocrine etiology. This aligns with geriatric principles: small metabolic deviations may herald reversible disease, and detailed evaluation, rather than attribution to ageing, can meaningfully change outcomes.

Ultimately, this case reinforces that endocrine disorders such as hypothyroidism should remain in the differential diagnosis for unexplained cognitive or functional decline in older adults, even when biochemical abnormalities appear mild. Incorporating routine geriatric assessments alongside systematic diagnostic frameworks can prevent diagnostic overshadowing and restore independence, dignity, and quality of life for older patients.

## Conclusions

This case serves as a reminder that functional decline in older adults is not always inevitable. Careful evaluation of hyponatremia may uncover potentially reversible contributors, such as hypothyroidism, leading to meaningful clinical improvement. A vigilant, geriatric-focused approach can help restore function and independence by identifying treatable conditions that might otherwise be attributed to ageing.
